# In Vitro Maturation of Fully Grown Mouse Antral Follicles in the Presence of 1 nM 2-Hydroxyestradiol Improves Oocytes’ Developmental Competence

**DOI:** 10.1007/s43032-020-00276-6

**Published:** 2020-08-05

**Authors:** Valeria Merico, Mario Zanoni, Alexis Parada-Bustamante, Silvia Garagna, Maurizio Zuccotti

**Affiliations:** 1 Laboratorio di Biologia dello Sviluppo, Dipartimento di Biologia e Biotecnologie “Lazzaro Spallanzani”, University of Pavia, Via Ferrata, 9, 27100 Pavia, Italy; 2 Centre for Health Technologies (C.H.T.), University of Pavia, Via Ferrata, 9, 27100 Pavia, Italy; 3 Institute of Maternal and Child Research, School of Medicine, University of Chile, Santiago, Chile

**Keywords:** Oocyte developmental competence, 2-Hydroxyestradiol, First polar body, F-Actin cap, Meiotic spindle, Microtubule organisation centres

## Abstract

Cathecolestrogens are estradiol metabolites produced during folliculogenesis in the mammalian ovary. 2-Hydroxyestradiol (2-OHE_2_) is one of the most abundant although its role remains unknown. The aim of this study is to investigate whether the presence of 2-OHE_2_ during the germinal vesicle-to-metaphase II transition affects oocyte meiotic and preimplantation developmental competence. Mouse cumulus-oocyte complexes (COCs), isolated from fully grown antral follicles, were in vitro–matured (IVM) in the presence of 2-OHE_2_ (0.1, 1, 10 or 100 nM) for 6 or 15 h; then, their meiotic and developmental competence was evaluated using a number of cytological quality markers. With the exception of the highest dose (100 nM), the addition of 2-OHE_2_ to the IVM medium, did not alter, compared with untreated control, the frequency of oocytes that reached the MII stage. Instead, IVM in the presence of 1 nM 2-OHE_2_ highly increased the rate of preimplantation development and blastocyst quality. To understand whether this positive effect could be attributed to the events occurring during meiosis resumption, we analysed a number of specific cytological quality markers of the asymmetric division, such as PB-I volume and position, presence and extension of the cortical F-actin cap, meiotic spindle shape and area, and microtubule organisation centre localisation. The results highlighted how the presence of 1 nM 2-OHE_2_ significantly improved the overall cytological organisation required for a correct asymmetric division. Our results contribute a first step to acknowledge a potential role of this estradiol metabolite during the GV-to-MII transition, contributing to the acquisition of oocytes developmental competence.

**Electronic supplementary material:**

The online version of this article (10.1007/s43032-020-00276-6) contains supplementary material, which is available to authorized users.

## Introduction

The acquisition of the oocyte developmental competence occurs inside the ovarian follicle thanks to a continuous cross-talk between the germ cell and the surrounding cumulus and granulosa cells. The follicle somatic cell components contribute crucial factors required, through several signalling pathways and regulatory loops, to complete oocyte growth and meiotic maturation [1, 2].

The importance to preserve the communication between oocyte and surrounding granulosa cells becomes evident when cumulus-oocyte complexes (COCs) are isolated from fully grown antral follicles and in vitro cultured [3, 4]. In the mouse, the efficiency of COCs in vitro maturation (IVM) varies depending on both strain (i.e., outbred or inbred) [5] and culture conditions [6, 7], although the meiotic and developmental competence of metaphase II (MII) oocytes obtained in vitro remains lower compared with that of ovulated MII oocytes [8, 9].

In antral follicles, granulosa cells are the major source of oestrogen production. These steroid hormones, present in all vertebrates [10] and some invertebrates [11], are the primary female sex hormones with a key role in the control of female reproduction [12]. The estradiol (E_2_)-oestrogen receptor system is functional in maintaining oocyte meiotic arrest, and, in association with an increase of the luteinising hormone, a decrease in E_2_ downregulates cGMP and cAMP inducing meiotic resumption and the extrusion of the first polar body (PB-I) [13, 14]. Several studies included E_2_ in the medium used for COCs IVM at different concentrations and time windows, showing inhibition of meiosis resumption [14, 15] and chromosome aberrations [16]. During this window of E_2_ downregulation, hydroxylases actively metabolise this hormone producing biologically active metabolites named catecholestrogens [17, 18], primarily at C-2 (2-hydroxyestradiol, 2-OHE_2_) or C-4 (4-hydroxyestradiol, 4-OHE_2_) positions, but also 6α-, 6β-, 7α-, 12β-, 15α-, 15β-, 16α-, and 16β-OHE_2_ [19–21].

Catecholestrogens have been shown to play a role in folliculogenesis both in Fish and Mammals. In teleosts (i.e., catfish and zebrafish), 2-OHE_2_ has a crucial role as component of the gonadotrophin cascade of regulation of oocyte maturation and ovulation [22, 23]. In vitro maturation of intact catfish follicles in the presence of 5 μM 2-OHE_2_ increased significantly the frequency of oocytes attaining germinal vesicle breakdown (GVBD); instead, it did not change it when oocytes were cultured in the absence of surrounding follicle cells [24].

In the mammalian ovary, oestrogen hydroxylases are well-represented showing a 100-fold increase from small antral to fully grown follicles and corpora lutea [25]. The produced 2-OHE_2_ and 4-OHE_2_ metabolites are detected in the follicular fluid of antral follicles; they increase concentration during follicle maturation [26] and have an influence on the differentiation and proliferation of granulosa [27] and luteal [28] cells. Also, they have been shown to stimulate progesterone production in small follicles [29], to enhance cAMP production and, as a consequence, improve FSH activity [27]. When injected, 4-OHE2 induced ovulation in immature rats [30] and improved the overall implantation success rate [31].

Overall, the studies described above highlight several roles for catecholestrogens during follicle maturation, and suggest a link with the female gamete meiotic and developmental competence. The objective of this study was to investigate how the presence of 2-OHE_2_ during the germinal vesicle-to-metaphase II transition affects oocytes meiotic and preimplantation developmental competence.

The main aim of the present study was to explore the effects of one of 2-OHE_2_ at concentrations ranging from 0.1 to 100 nM, during IVM of mouse fully grown antral follicles from the GV to the MII stage. Oocytes that reached MII were inseminated with capacitated sperm and their preimplantation developmental competence recorded. Our results indicate a dose-dependent effect of 2-OHE_2_, that, when present in the IVM culture medium at 1 nM concentration, induces a significant beneficial effect on both meiotic resumption and preimplantation embryonic development.

## Materials and Methods

### Animals and Reagents

Four 6-week-old female and 6-month-old male CD1 mice were purchased from Charles River (Como, Italy). Animals were maintained under controlled room conditions (22 °C, with 60% air moisture and 12/12 light/dark photoperiod), and investigations were conducted in accordance with the guiding principles of European (n. 2010/63/UE) and Italian (n. 26/2014) laws protecting animals used for scientific research. All chemicals used were purchased from Sigma-Aldrich (St. Louis, MO, USA), unless otherwise stated. Ultrapure water used for preparing media and solutions was obtained with a MilliQ® IQ 7000 system (Merck) equipped with a Biopak® Ultrafiltration filter (Merck).

### COC Isolation and In Vitro Maturation

Females were injected with 5 I.U. Folligon (Intervet Srl, Italy), and 48 h later, COCs were isolated from the ovary. Briefly, ovaries were placed in 1 ml HEPES-buffered isolation medium: α-MEM plus GlutaMAX (Life Technology; cat.no. 32561–029) supplemented with 6 mg/ml HEPES, 5% fetal bovine serum (FBS), 0.23 mM sodium pyruvate, 1 mg/ml Fetuin, 100 I.U./ml penicillin and 75 μg/ml streptomycin in a 30-mm Petri dish (Corning, Euroclone, Italy) under a stereomicroscope. Fully grown antral follicles were punctured from the ovarian surface with a thin and sharp-pulled sterile Pasteur pipette. Only intact COCs with layers of cumulus cells completely surrounding the oocyte were used in further experiments. After washing in fresh HEPES-buffered isolation medium, COCs were cultured in 100 μl drops of α-MEM supplemented with 5% FBS; 0.23 mM sodium pyruvate; 1 mg/ml fetuin; 100 I.U./ml penicillin and 75 μg/ml streptomycin; 50 mI.U./ml FSH; and 10 ng/ml epidermal growth factor (EGF) in the absence (IVM) or presence (IVM-2-OHE_2_) of 0.1 nM, 1 nM, 10 nM or 100 nM 2-OHE_2_ (prepared in dimethyl-sulfoxide, DMSO, at a maximum concentration of 0.002%. As reported earlier [32] and confirmed by our results (Tables 1S and 2S), the presence of DMSO in IVM medium, at concentrations up to 0.1%, did not have adverse effects on oocyte maturation and on further preimplantation development. Thus, DMSO was always present in our IVM medium, independently on the presence or absence of 2-OHE_2_.

IVM drops were layered with mineral oil and placed in an incubator at 37 °C, 100% humidity in 5% CO_2_, 5% O_2_ and 90% N_2_ for 6 h (MI stage) or 15 h (MII stage). At the end of the culture period, MI or MII (confirmed by the presence of PB-I) oocytes were used for further experiments.

In order to collect in vivo–matured oocytes, females were injected with 5 I.U. Folligon followed, 48 h later, by 5 I.U. Corulon (Intervet) and, after 6 h (MI) or 15 h (MII), gametes were isolated from ovarian surface or from the oviducts, respectively.

MII oocytes, with the exception of those used for in vitro fertilisation (IVF), were denuded of their cumulus cells by a brief treatment with 500 I.U. hyaluronidase.

### In Vitro Fertilisation and Preimplantation Development

MII oocytes surrounded by their cumulus cells were inseminated with capacitated sperm (2 × 10^6^ sperm/ml) as previously described [33]. Two hours after insemination, oocytes were transferred into 40 μl drops of Whittigham medium (2 μl/oocyte) for an additional hour. Then, presumptive zygotes (as determined by the presence of a second polar body, PB-II) were transferred into 40 μl drops of M16 medium (2 μl/oocyte) supplemented with 0.4% BSA, 2 mM glutamine (Gibco), 5 mM taurine and 0.23 mM pyruvate for preimplantation development. Embryonic developmental rate was evaluated at 24 (2-cell stage), 48 (4-cell) and 96 (blastocyst) hours post-insemination.

### Immunofluorescence

For the analysis of the meiotic spindle, MI or MII oocytes were fixed in 4% paraformaldehyde (PFA) in a microtubule stabilisation buffer (137 mM NaCl, 5 mM KCl, 1.1 mM Na_2_HPO_4_, 0.4 mM KH_2_PO_4_, 2 mM MgCl_2_, 4 mM NaHCO_3_, 2 mM EGTA, 5 mM PIPES, 5.5 mM Glucose, 0.1 M Glycine) containing 1% Triton X-100 for 45 min at 37 °C in agitation. Fixed oocytes were washed 3 times, 5 min each, in a washing solution (WS: 1x PBS, 0.2% sodium azide; 0.2% powdered milk, 2% FBS, 1% BSA, 0.1% glycine) supplemented with 0.2% Tween 20 and stored at 4 °C in WS.

Double labelling of α- and γ-tubulin, raised in the same species, was done through sequential detection of both epitopes, i.e. primary and then secondary antibodies for the first epitope followed by primary and then secondary antibodies for the second epitope.

Oocytes were incubated with a mouse anti-α-tubulin antibody (cat. N. T9026; dilution 1:1000 in WS) for 1 h at 37 °C in agitation. Then, gametes were washed 3 times, 15 min each, in WS at room temperature in agitation and incubated with the secondary AlexaFluo488 goat anti-mouse IgG antibody (cat. N. A11001, Molecular Probes; dilution 1:1000); then the gametes were incubated with a mouse anti-γ-tubulin antibody (cat. N. T5326; dilution 1:1000 in WS) for 1 h at 37 °C in agitation, washed 3 times, 15 min each, in WS at room temperature in agitation and simultaneously incubated with the secondary AlexaFluo633 goat anti-mouse IgG antibody (cat. N. A21053, Molecular Probes; dilution 1:1000) and TRITC-conjugated phalloidin (cat. N. P1951; dilution 1:1000 in WS). After incubation, oocytes were washed 3 times, 15 min each, in WS, counterstained with 0.2 μg/ml 4′,6-diamidino-2-phenylindole dihydrochloride (DAPI; cat N. D8417) and mounted in Vectashield (cat. N. H-1000, Vector Laboratories).

Ninety-six hours post-insemination, blastocysts were fixed in freshly prepared 4% PFA for 30 min and then washed in 1x PBS containing 0.1% Tween 20 (PBT). Embryos were processed for immunolabelling using a rabbit polyclonal anti-human OCT4 (cat. N. ab19857, Abcam; diluted 1:400 in WS) or a rabbit anti-human CDX2 (cat. N. 3977, Cell Signaling Technology; diluted 1:100 in WS) antibodies. Blastocysts were incubated with primary antibodies for 1 h at 37 °C and then washed twice (30 min each) in WS under gentle agitation. Then, they were incubated with AlexaFluor555 goat anti-rabbit IgG (cat. N. A21428, Molecular Probes; diluted 1:500 in WS) secondary antibody for 1 h at 37 °C, followed by 2 washes (30 min each) under gentle agitation, counterstained with DAPI (0.2 μg/ml in PBS, 10 min) and mounted in Vectashield.

Samples were examined using a Leica TCS SP8 confocal microscope equipped with lasers for AlexaFluor488, AlexaFluor555, AlexaFluor633 and DAPI fluorochromes. Stacks were obtained with axial distances of 0.3 μm. Immunolocalisation of OCT4 and CDX2 on blastocysts was analysed with an Olympus BX60 fluorescence microscope (Olympus, Ibaraki, Japan) equipped with single-bandpass filters for DAPI, AlexaFluor488 and AlexaFluor555; images were captured with a DP72 camera (Olympus) controlled by the CellSens Dimension 1.4.1 software (Olympus) and processed with Image*J* (http://imagej.nih.gov/ij/).

### First Polar Body Morphology and Size

CCs-free MII oocytes were photographed using an inverted Olympus IX71 microscope equipped with a JVC KY-F58 3-CCD camera and a micromanipulator. For each oocyte, images (under × 2x objective) were captured with a frontal and lateral PB-I orientation obtained moving the oocytes using the micromanipulator micropipette. Only intact PB-Is were considered for further analyses. Pictures were analysed using the CellSens Dimension 1.4.1 software and the PB-I volumes calculated drawing three lines corresponding to the three radii of each PB-I.

### Amplitude Angle Between PB-I and Oocyte Chromosomes

CCs-free MII oocytes were stained with 0.05 μg/ml bisBenzimide H-33342 trihydrochloride (Hoechst 33342; cat. N. D2261) in M2 medium for 15 min. Then, by using an inverted Olympus IX71 fluorescence microscope equipped with a micromanipulation system, single oocytes were rotated until metaphase chromosomes of both PB-I and oocyte appeared on the same focus. Pictures were taken, under × 20 objective, with a JVC KY-F58 3-CCD camera and analysed using the CellSens Dimension 1.4.1 software. Two lines were drawn from the centroid of the oocyte towards either the oocyte’s or PB-I’s chromosomes and the resulting angle was measured.

### F-Actin Cap Intensity Profile and Amplitude Angle

The F-actin cap intensity was calculated, on the phalloidin red channel image, using the ImageJ software. A line was drawn (see Fig. 3) that, from the cortical region (p), intersected the MI or MII chromosomes, passed through the centroid (c) and reached the opposite pole (d). The fluorescence intensity was measured as p/d ratio. MI or MII oocytes of the three experimental conditions were divided into two groups: group I, oocytes with intensity (I) comprised between 1.8 < I ≤ 21.8; and group II, 1.1 < I ≤ 1.8.

The amplitude angle of the F-actin cap was determined drawing two lines starting from the centroid of the oocyte and the ends of the F-actin cap defined by phalloidin staining (see Fig. 4a).

### Spindle Area and Shape

Using the CellSens Dimension software, the spindle area, identified by the α-tubulin signal, was calculated considering the spindle as a barrel made of two trapezoids with a common side at the equator width, the shorter sides at the two spindle poles and the height as half the length between the two poles (Fig. 5).

Based on the ratio between the equator width and the mean of the widths at the two poles, a barrel and a rectangular configuration were observed.

### MTOC Distribution

The microtubule organisation centre (MTOC) distribution was established based on the γ-tubulin signal. Two different patterns were identified: (i) a single focus, in which all the γ-tubulin signals were distributed as a single cluster, localised at each spindle pole; and (ii) multiple foci, in which the γ-tubulin signals were distributed as two or more clusters along the width of the two poles.

### Statistical Analysis

Statistical analyses were carried out using the Sigma Stat 3.5 software. Data, obtained from at least five independent experiments, were analysed by the Student’s *t* test for comparing two conditions or by the one-way ANOVA for comparing more than two groups. In the presence of significant differences, one-way ANOVA test was followed by the Fisher LSD Method (post hoc test). When data were not normally distributed, statistical analysis was performed either by the Mann-Whitney test (when the comparison involved two samples) or by the Kruskal–Wallis test together with the Dunn’s multiple range test (for the comparison of more than two samples). Parametric and nonparametric data were expressed as the error standard ± mean (SEM). The analysis of the absolute frequencies was performed using a Fisher’s exact test*.* Differences were considered significant for *p* values ≤ 0.05.

## Results

To test the effects of 2-OHE_2_ on oocyte developmental competence, COCs were in vitro–matured for 15 h in the presence of 0.1, 1, 10 or 100 nM 2-OHE_2_.

### Oocytes In Vitro Maturation

Based on the presence of the PB-I, more than 90% of oocytes in all five experimental conditions reached the MII phase. Compared with the control in vitro–matured COCs (IVM) (Table 1), oocytes whose COCs were cultured in the presence of 2-OHE_2_ (IVM-2-OHE_2_), spontaneously resumed meiosis and reached MII without significant differences (*p* > 0.05), with the exception of those treated with the highest 2-OHE_2_ concentration (100 nM) in which a decreased MII rate was observed (90.4 ± 1.5%; *p* = 0.015). This latest experimental group also showed significantly higher frequency of fragmented oocytes (3.1 ± 1.4%; *p* = 0.002).

**Table 1:** Rate of in vitro maturation and preimplantation embryonic development of COCs matured in the presence or absence of 2-OHE_2_

Treatment	Stages of oocyte maturation and preimplantation development % ± SEM (number)*							
	COCs	Blocked at GV or GVBD	Fragmented or picnotic	MII	Inseminated MII	2-cell	4-cell**	Blastocyst**
OV	n.d.	n.d.	n.d.	n.d.	145	87.7 ± 3.6^a^ (125)	91.4 ± 2.8^a^ (89)	84.2 ± 3.9^a^ (80)
IVM	670	5.1 ± 0.8 (32)	0.4 ± 0.2^a^ (3)	94.6 ± 0.8^a^ (635)	421	64.7 ± 2.1^b^ (273)	55.5 ± 2.8^b^ (149)	28.6 ± 1.6^b^ (76)
IVM-2-OHE_2_								
0.1 nM	193	4.3 ± 0.6 (8)	1.2 ± 0.7^a^ (2)	94.5 ± 1.0^a^ (183)	174	68.5 ± 2.5^b^ (120)	67.8 ± 3.5^c^ (79)	32.4 ± 4.7^b^ (37)
1 nM	512	3.2 ± 0.6 (16)	0.8 ± 0.5^a^ (3)	95.8 ± 0.8^a^ (492)	443	65.8 ± 3.9^b^ (288)	68.4 ± 1.7^c^ (146)	49.6 ± 1.2^c^ (143)
10 nM	145	6.0 ± 0.8 (9)	1.3 ± 0.9^a^ (2)	92.6 ± 1.6^a^ (134)	123	68.4 ± 2.0^b^ (84)	65.3 ± 2.2^c^ (55)	47.8 ± 4.9^c^ (42)
100 nM	163	6.5 ± 0.9 (11)	3.1 ± 1.4^b^ (9)	90.4 ± 1.5^b^ (143)	137	67.6 ± 3.1^b^ (92)	62.5 ± 4.8^b^ (58)	29.2 ± 1.6^b^ (27)

*In the same column, different superscript letters indicate a significant difference

**The developmental rate was calculated based on the number of 2-cell embryos (100%)

### MII Oocyte Developmental Competence

Following in vitro insemination, MII oocytes of the five experimental groups reached the 2-cell stage with the same rate (*p* = 0.884) (Table 1). However, those treated with 2-OHE_2_ attained the 4-cell stage with significantly higher frequency (≥ 65.3%; *p* ≤ 0.047) compared with IVM (55.5%), with the exception of the highest 2-OHE_2_ dose (100 nM) in which the increment was not significant (62.5 ± 5.0%, *p* = 0.148). Compared with IVM (28.6 ± 1.6%), 32.4 ± 4.7% (*p* = 0.283), 49.6 ± 1.2% (*p* < 0.001), 47.8 ± 4.9% (*p* < 0.001) and 29.2 ± 1.6% (*p* = 0.875) of 0.1 nM, 1 nM, 10 nM or 100 nM IVM-2-OHE_2_-treated embryos reached the blastocyst stage, respectively.

Overall, these results indicate a highly significant improvement of the preimplantation developmental rate to blastocyst of those antral oocytes whose COCs were matured in the presence of 1 or 10 nM 2-OHE_2_. Comparison between ovulated MII oocytes (OV^MII^) and IVM or IVM-2-OHE_2_ showed that the former performed always significantly better (Table 1).

### Blastocyst Cell Number

The quality of blastocysts obtained with the 1 or 10 nM 2-OHE_2_ experimental conditions was evaluated based on their total number of cells and on the number of trophectoderm (TE) or inner cell mass (ICM) blastomeres, compared with control IVM blastocysts or to those derived from OV^MII^. Embryos were processed for the immunofluorescence localisation of OCT4 and CDX2 proteins, markers of ICM and TE cells, respectively. As shown earlier [9], the total cell number of blastocysts derived from IVM-COCs (32.3 ± 1.6) was significantly lower than that of control OV^MII^ oocytes (52.5 ± 1.0; *p* < 0.001) (Table 2). Compared with IVM blastocysts, the presence of 1 nM 2-OHE_2_ during COC maturation significantly (*p* = 0.011) increased the number of cells (43.2 ± 4.6), instead that of 10 nM IVM-2-OHE_2_ (34.4 ± 1.7) was not significantly different (*p* = 0.561). This higher number of cells could be attributed to an increased number of ICM blastomeres (9.9 ± 0.7 vs. 7.4 ± 0.6; *p* = 0.003).

**Table 2:** Number ± SEM (*) of blastomeres forming the whole blastocyst, the inner cell mass (OCT4) or the trophectoderm (CDX2)

Treatment	No. of blastocysts analysed	DAPI	OCT4	CDX2
OV	18	52.5 ± 1.0^a^	13.2 ± 0.6^a^	39.3 ± 1.0^a^
IVM	19	32.3 ± 1.6^b^	7.4 ± 0.6^b^	25.3 ± 1.4^b^
IVM-2-OHE_2_				
1 nM	18	43.2 ± 4.6^c^	9.9 ± 0.7^c^	33.6 ± 4.1^a^
10 nM	21	34.4 ± 1.7^b^	8.4 ± 0.4^b,c^	26.0 ± 1.5^b^

(*): In the same column, different superscript letters indicate a significant difference

In summary, the results described highlight a significant improvement of the preimplantation developmental rate when COCs were matured in the presence of 1 nM or 10 nM 2-OHE_2_, and better blastocyst quality with 1 nM concentration.

Next, we addressed the question of whether this observed improvement in the presence of 1 nM 2-OHE_2_ could be attributed to the latest events of meiotic resumption, when the first asymmetric division occurs culminating with PB-I extrusion. To this end, we employed a number of known cytological quality markers of the meiotic division to compare MI or MII oocytes matured in vitro, either in the absence or in the presence of 1 nM 2-OHE_2_, and those obtained by ovulation. Specifically, we analysed (i) the PB-I volume [6, 34–43] and its position in respect to the oocyte’s spindle [44]; (ii) the presence and extension of the cortical F-actin cap [45–47]; (iii) the meiotic spindle shape and area [48–51] and (iv) the microtubule organisation centre (MTOC) localisation [8, 48, 52].

### PB-I Volume and Position

**Fig. 1:**
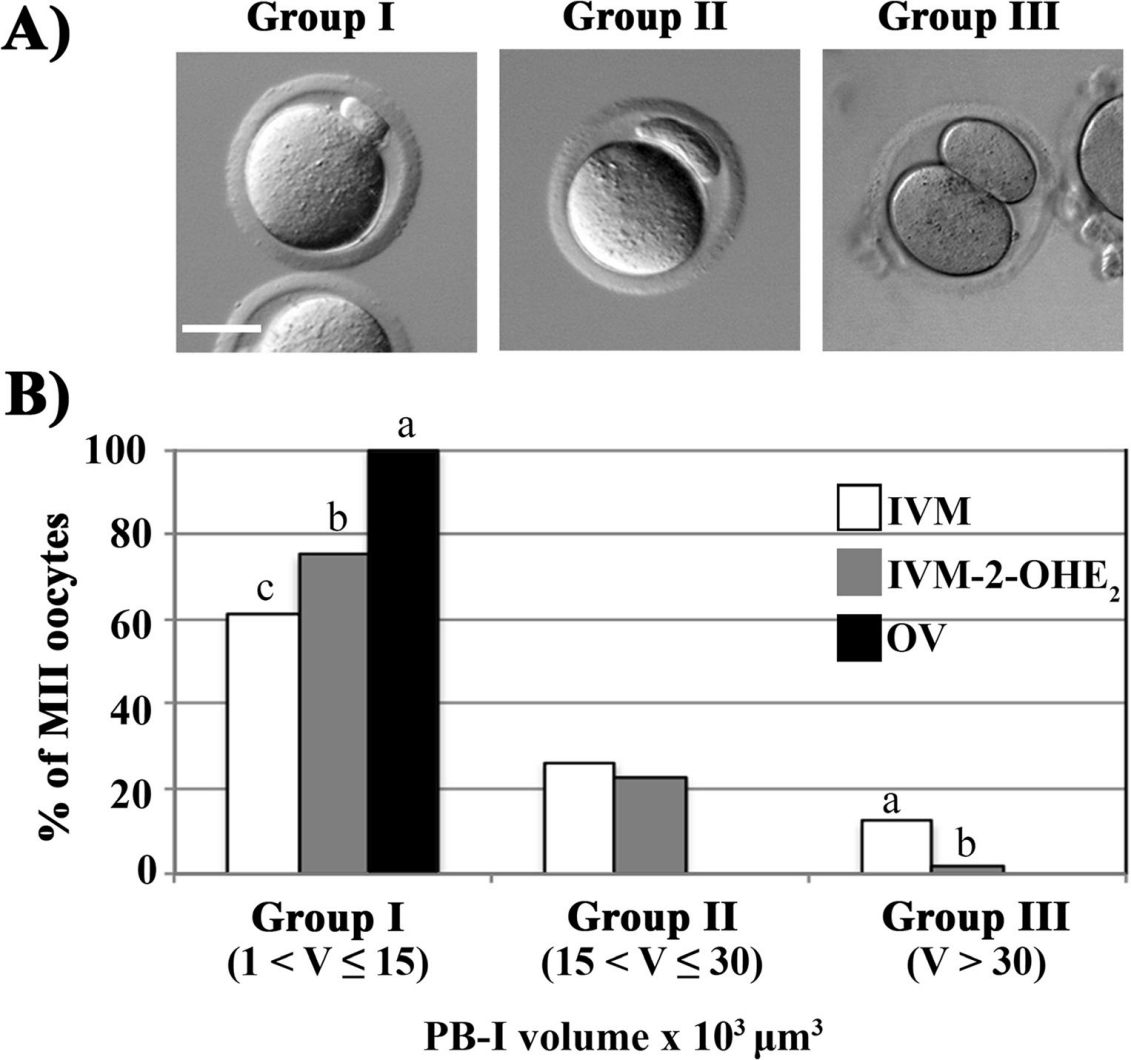
PB-I size. MII oocytes from the in vitro–maturated (IVM), 1 nM IVM-2-OHE_2_ or ovulated (OV) groups were analysed to determine the PB-I volume (V) and divided (absolute percentage) into three groups (**a**): group I, with a volume comprised between 1 × 10^3^ < V ≤ 15 × 10^3^ μm^3^; group II, 15 × 10^3^ < V ≤ 30 × 10^3^ μm^3^ and group III, V > 30 × 10^3^ μm^3^. (**b**) Following treatment with 2-OHE_2_, we observed a significant increase of MII oocytes with a correct group I PB-I size, although it remained lower compared with that of OV^MII^. In the same group, different superscript letters indicate the presence of a significant difference. Bar, 40 μm

Based on the PB-I volume, we identified the presence of three main MII oocytes groups: group I, with a volume comprised between 1 × 10^3^ and 15 × 10^3^ μm^3^; group II, between 15 × 10^3^ and 30 × 10^3^ μm^3^ and group III, > 30 × 10^3^ μm^3^ (Fig. 1).

Whilst all OV^MII^ (*p* ≤ 0.001) oocytes belonged to group I, 60.6% and 75.2% of IVM^MII^ and IVM-2-OHE_2_^MII^, respectively, the remaining 26.8% or 12.6% (IVM^MII^) and 23.0% or 1.8% (IVM-2-OHE_2_^MII^) were classified in group II (*p* = 0.601) or III (*p* = 0.003), respectively. Group III oocytes appeared similar to a 2-cell embryo, indicating the occurrence of an almost symmetrical division.

**Fig. 2:**
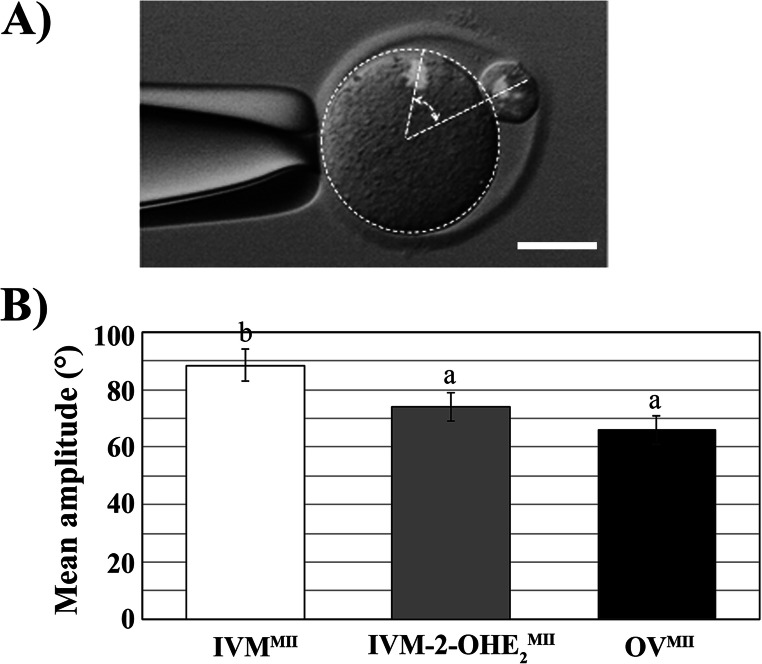
PB-I position relative to MII spindle. (**a**) Photograph of a fixed oocyte observed with bright-field and fluorescence, showing the relative position of PB-I to MII spindle. The chromosomes were stained with DAPI. The angle between the lines drawn from the centroid to the PB-I chromosomes or the oocyte spindle was measured and (**b**) the mean amplitude (± SEM) was calculated in the three experimental groups. Different superscript letters indicate the presence of a significant difference. The 2-OHE_2_ treatment improved significantly the positioning of the MII plate erasing the difference existing between IVM^MII^ and OV^MII^. Bar, 30 μm

The PB-I position was calculated as the amplitude of the angle measured at the oocyte’s centroid between oocyte’s and PB-I’s chromosomes (Fig. 2a). IVM-2-OHE_2_^MII^ oocytes showed significantly smaller angle compared with IVM^MII^ (*p* = 0.044) oocytes, but similar (*p* = 0.399) to that measured in OV^MII^ oocytes (Fig. 2b).

### Presence and Extension of the Cortical F-Actin Cap

**Fig. 3:**
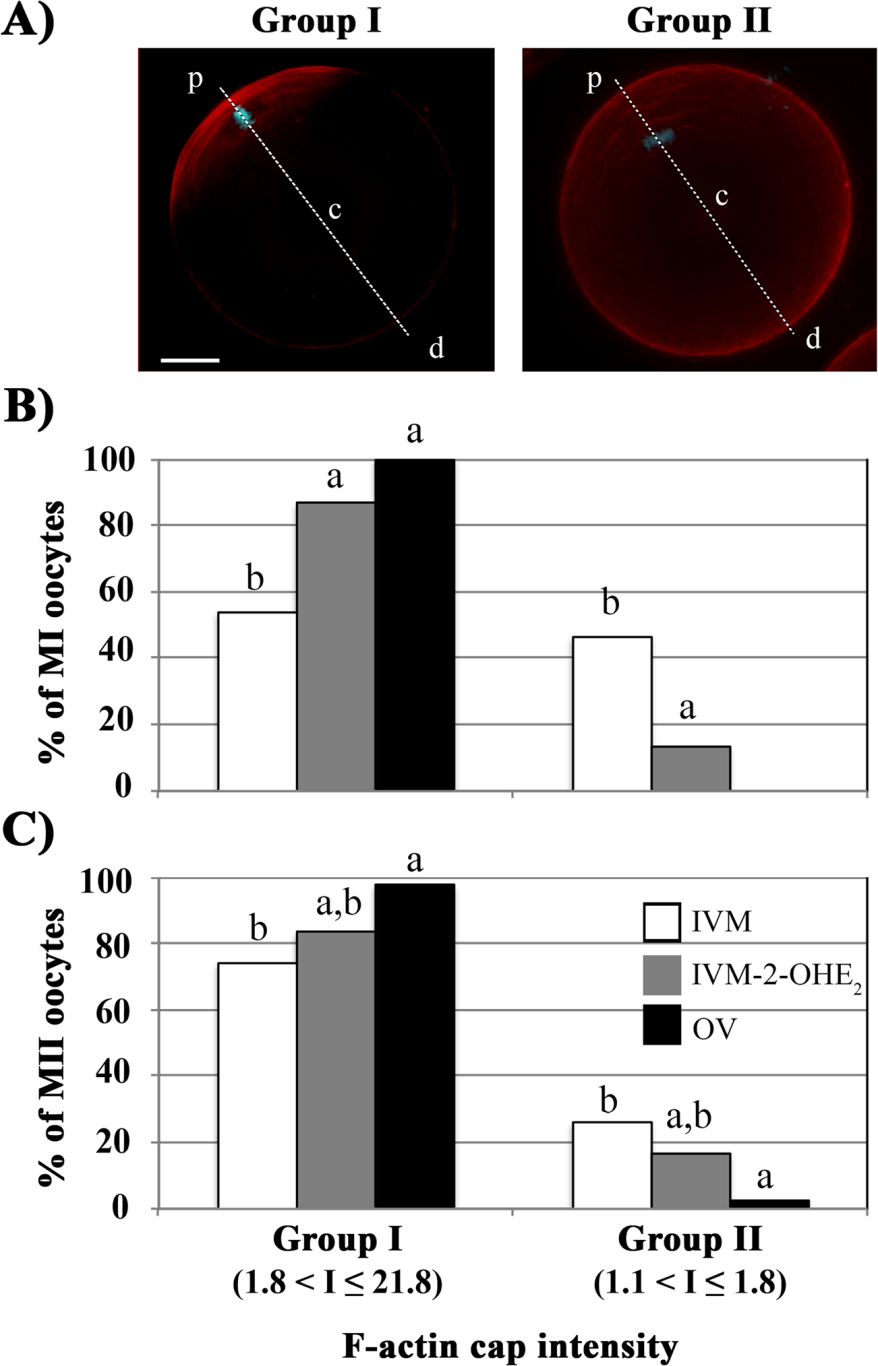
Relative fluorescence intensity of the F-actin cap. The F-actin cap intensity was calculated, on the phalloidin red channel image, drawing a line that, from the cortical region (p), intersects the MI or MII chromosomes, passes through the centroid (c) and reaches the opposite pole (d). The fluorescence intensity was measured as p/d ratio using the Image*J* software. MI or MII oocytes of the three experimental conditions were divided into two groups (**a**): group I, oocytes with an intensity (I) comprised between 1.8 < I ≤ 21.8; and group II, 1.1 < I ≤ 1.8. Following 2-OHE_2_ treatment, the matured MI (**b**) and MII (**c**) oocytes (absolute percentage) displayed an F-actin fluorescent intensity comparable with that observed in OV^MI^ or OV^MII^ oocytes. Different superscript letters indicate the presence of a significant difference. Bar, 20 μm

The eccentric position of the metaphase spindle and the consequent asymmetric meiotic division lead to the formation of two daughter cells with very different volumes. A crucial event during this cortical polarisation is the formation of a thick F-actin cap at the site of PB, just prior to its extrusion. Oocytes from OV, IVM or IVM-2-OHE_2_ groups were labelled with phalloidin after 6 h (MI) or 15 h (MII) of maturation. Based on the fluorescence intensity of the F-actin cap, we classified two main groups of oocytes (Fig. 3a): group I with an intensity comprised between 1.8 and 21.8; and a much fainter, group II, comprised between 1.1 and 1.8. All OV^MI^ belonged to group I, compared with 53.8% (*p* < 0.001) of IVM^MI^ oocytes, whilst the remaining 46.2% IVM^MI^ oocytes showed a fainter, group II, F-actin cap (Fig. 3b). The presence of 2-OHE_2_ in the culture medium significantly increased (*p* = 0.016) to 86.7%; the number of MI oocytes with a clearly visible F-actin cap was not significantly (*p* = 0.112) different compared with that of OV^MI^ oocytes (Fig. 3b). Similarly, the frequency of IVM-2-OHE_2_^MII^ oocytes with marked F-actin cap (83.3%) resulted not significantly different (*p* = 0.059) compared with that of OV^MII^ oocytes (97.5%) (Fig. 3c).

**Fig. 4:**
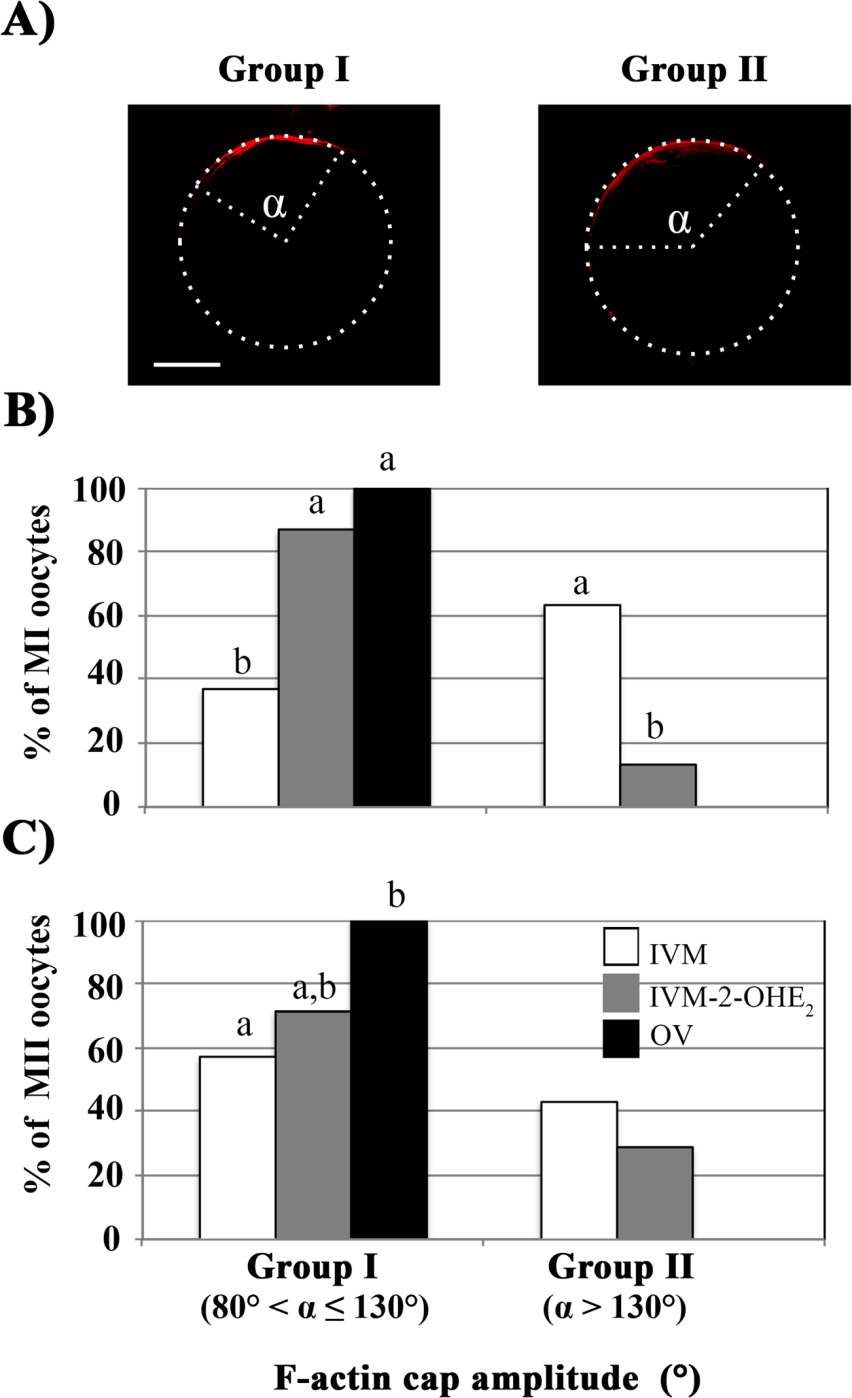
F-actin cap extension. The extension of F-actin cap was measured as amplitude of the angle comprised between the two lines drawn from the centroid of the oocyte and the ends of the F-actin cap. MI or MII oocytes of IVM, IVM-2-OHE_2_ and OV were divided into two groups (**a**). Group I, oocytes with an angle amplitude (*α*) values 80° < *α* ≤ 130°; group II, oocytes with *α* values *α* > 130°. The MI or MII oocytes (absolute percentage) of IVM-2-OHE_2_ showing an F-actin extension comparable with that observed in OV oocytes (**b**–**c**). Different superscript letters indicate the presence of a significant difference. Bar, 20 μm

Then, we measured the amplitude of the angle formed between the oocyte centroid and the cortical F-actin cap ends in both in vitro– and in vivo–matured oocytes (Fig. 4a). Whilst all OV^MI^ showed an angle comprised between 80° and 130°, 63.3% (*p* < 0.001) of IVM^MI^ oocytes displayed an angle > 130° (Fig. 4b). When cultured in the presence of 2-OHE_2_, the majority (88.5%) of IVM-2-OHE_2_^MI^ oocytes showed an angle similar to that of OV^MI^ (*p* = 0.105; Fig. 4b), whereas only 11.5% oocytes had a wider (> 130°) angle. The same analysis performed on MII oocytes gave similar results (Fig. 4c).

### Meiotic Spindle Shape and Area

Oocytes from the three experimental groups were fixed after 6 h (MI) or 15 h (MII) of maturation, immunolabelled with anti-alpha tubulin to visualise their spindle and counterstained with DAPI.

**Fig. 5:**
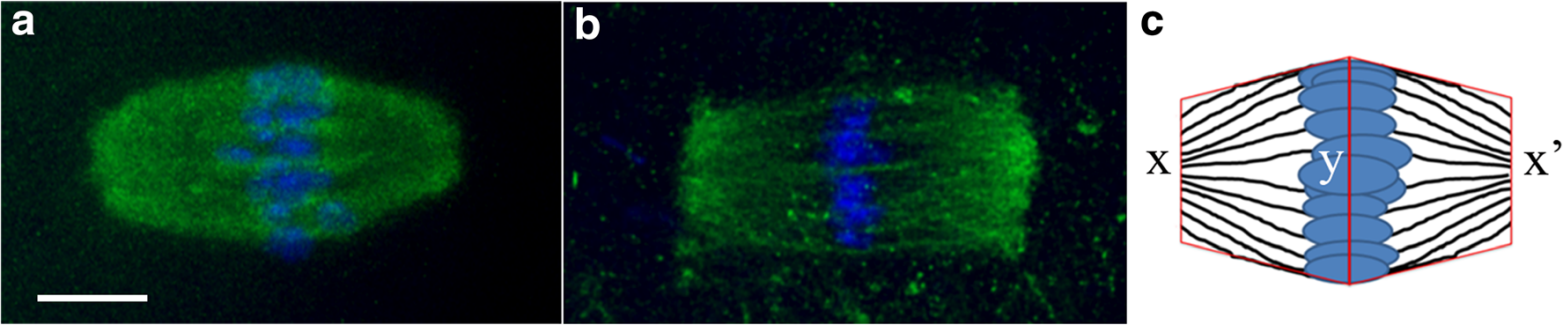
Meiotic spindle morphology. Representative fluorescent images of MI spindles with barrel- (**a**) or rectangular-shape (**b**), labelled with α-tubulin (green) and counterstained with DAPI (blue). Bar = 6 μm. (**c**) The spindle area was calculated as the sum of the areas of the two drawn trapezoids with a common side at the equator (y) and the shorter sides at the two spindle poles (x, x′)

The shape of the metaphase spindle was calculated as the ratio between the central width (Fig. 5a, y) and the mean between the width at the two poles (Fig. 5a, x and x′). Two main spindle shapes were observed: a barrel-like spindle (Fig. 5b) with a ratio value ≥ 1.8 and a rectangular-like spindle (Fig. 5c) with a ratio value < 1.8.

All the OV^MI^ oocytes displayed a barrel-like shape with an area of 173.6 ± 8.6 μm^2^ (Table 3). When compared with IVM^MI^, the latter showed a significant (*p* = 0.004) decreased frequency (75%) of oocytes with a barrel-like spindle. The maturation of COCs in the presence of 2-OHE_2_ decreased the frequency of oocytes with a rectangular-like spindle to values not significantly different (13.3%; *p* = 0.077) compared with that of OV^MI^.

**Table 3:** MI or MII oocytes showing a metaphase plate with a barrel- or rectangular-shape and their size in area (μm^2^)

		IVM		IVM-2-OHE_2_		OV	
		% (N.)	Area (mean ± SEM)	% (N.)	Area (mean ± SEM)	% (N.)	Area (mean ± SEM)
MI	Barrel	75.0^a^ (30/40)	334.4 ± 17.5^a^	86.7^a,b^ (39/45)	235.0 ± 14.4^b^	100^b^ (36/36)	173.6 ± 8.6^c^
	Rectangular	25.0 (10/40)	277.9 ± 26.8	13.3 (6/45)	271.85 ± 7.0	n.d.	n.d.
MII	Barrel	53.8^a^ (28/52)	132.2 ± 6.2	81.0^a,b^ (34/42)	122.0 ± 7.7	100 (40/40)	118.6 ± 5.1
	Rectangular	46.2 (24/52)	127.1 ± 5.4	19.0 (8/42)	120.2 ± 1.8	n.d.	n.d.

In the same raw, different superscript letters indicate a significant difference

Similarly, all OV^MII^ oocytes showed a barrel-like spindle, whereas the two IVM conditions produced both barrel- and rectangular-like spindles. Again, IVM in the presence of 2-OHE_2_ gave a significantly (*p* = 0.011) higher frequency (81%) of oocytes with barrel-like spindle compared with those matured in the absence of the hormone (53.8%).

### MTOC Localisation

**Fig. 6:**
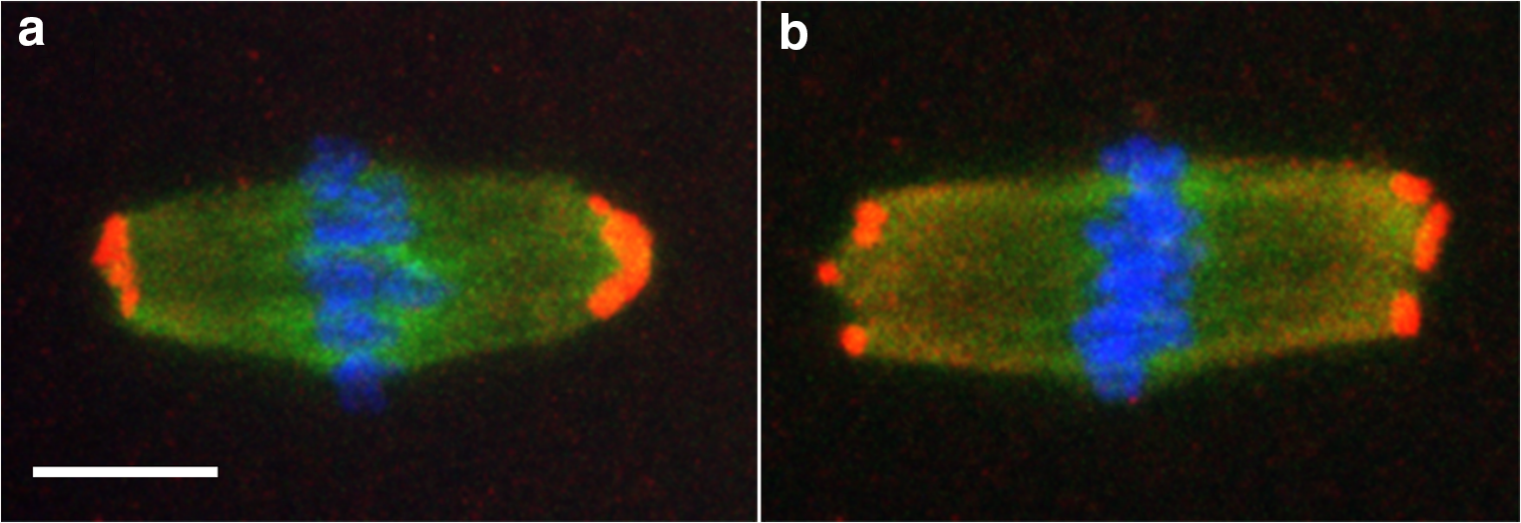
MTOC localisation. Representative fluorescent images of MTOCs localisation in MI spindles with barrel- (**a**) or rectangular-shape (**b**). Double immunostaining with α- (green) and γ- (red) tubulin, the latter displaying two main patterns of MTOCs localisation: one with all MTOCs clustered (**a**), the other, with MTOCs dispersed (**b**) at the spindle poles. Chromosomes are counterstained with DAPI (blue). Bar, 6 μm

Gamma-tubulin, a component of the microtubule organisation centres (MTOCs), plays a key role in spindle formation during meiosis [53]. Here, we analysed the MTOCs localisation in MI and MII oocytes obtained from the three experimental conditions. Double immunostaining with α- and γ-tubulin showed two main patterns of MTOCs localisation: one with all MTOCs clustered at each of the two spindle poles (single signal) (Fig. 6a); the other, with the MTOCs dispersed at the two spindle poles (multiple signals) (Fig. 6b).

The great majority of control OV^MI^ (97.2%) or OV^MII^ (93.3%) oocytes displayed only a single signal pattern of MTOC localisation associated to a barrel-like spindle (Table 4). Instead, only 33.3% of IVM^MI^ or 14.3% IVM^MII^ oocytes showed the combination of a barrel-like spindle and a single MTOC signal. These frequencies increased significantly when COCs were matured in the presence of 2-OHE2 with 57.9% (*p* = 0.039) of IVM-2-OHE_2_^MI^ and 57.1% (*p* < 0.001) of IVM-2-OHE_2_^MII^ displaying a barrel-like spindle and a single MTOC signal.

**Table 4:** Percentage (number) of MI or MII oocytes with clustered or dispersed MTOCs associated with their spindle poles (*)

		IVM		IVM-2-OHE_2_		OV	
		Clustered	Dispersed	Clustered	Dispersed	Clustered	Dispersed
MI	Barrel	33.3^c^ (16/48)	29.2^b^ (14/48)	57.9^b^ (22/38)	10.5^a^ (4/38)	97.2^a^ (35/36)	2.8^a^ (1/36)
	Rectangular	6.3 (3/48)	31.5 (15/48)	7.9 (3/38)	23.7 (9/38)	n.d.	n.d
MII	Barrel	14.3^c^ (8/56)	21.4 (12/56)	57.1^b^ (24/42)	14.3 (6/42)	93.3^a^ (28/30)	6.7 (2/30)
	Rectangular	14.3 (8/56)	50.0^b^ (28/56)	4.8 (2/42)	23.8^a^ (10/42)	n.d.	n.d

*In the same raw, different superscript letters indicate a significant difference. Statistical analysis was done comparing, separately, either clustered or dispersed MTOCs amongst the three experimental conditions

## Discussion

**Fig. 7:**
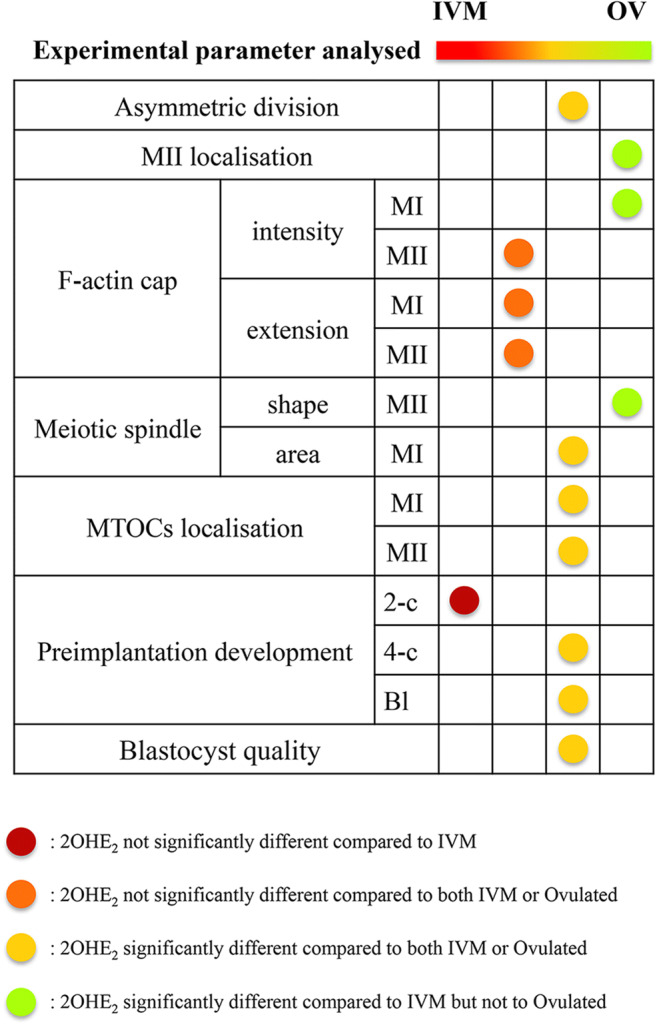
Graphical summary of the results highlighting the improvement of the meiotic and developmental competence of oocytes matured in the presence of 1 nM 2-OHE_2_ when compared with oocytes matured in the absence of the catecholestrogen (IVM) and towards the acquisition of properties pertaining to ovulated oocytes (OV)

This study demonstrates a dose-dependent response to the presence of 2-OHE_2_ during the GV-to-MII transition on the oocyte meiotic and developmental competence (Fig. 7). Although the overall quality of oocytes cultured in the presence of 2-OHE_2_ remained lower compared with that of control OV oocytes, the presence of this catecholestrogen clearly produced a highly significant improvement when compared with COCs that were matured in the absence of 2-OHE_2_. Our results show that whilst the rate of meiotic resumption remained unchanged at doses comprised between 0.1and 10 nM, it decreased significantly at the highest dose employed. It was the combined observation of the developmental rate and blastocyst quality that highlighted the highly significant improvement obtained following GV-to-MII maturation in the presence of 1 nM dose, with a sharp 21% increase of embryos that completed preimplantation development. Furthermore, these blastocysts displayed higher number of blastomeres, a sign of an intensified cell cycle rate [9, 54, 55].

This improved developmental competence contradicts earlier studies with bovine COCs that, when matured in the presence of 3 μM of this catecholestrogen, display a 5-fold decrease of development to blastocyst [56]. Also, COC maturation in the presence of a 2-OHE_2_ metabolite (2-methoxyestradiol, 2-ME_2_) caused spindle aberrations, chromosome congression failure and non-disjunction at the time of mouse oocytes meiosis resumption [57] and had negative effects on bovine oocyte developmental competence [56]. The improved results we obtained might find an explanation in the doses used in our experiments (0.1–100 nM), which are closer to the 15 nM physiological condition described in pig preovulatory follicles [26].

To understand the reason of the recorded oocyte developmental competence improvement, we used a number of known cytological quality markers of meiotic resumption. All these markers showed that the presence of 2-OHE_2_ smoothed the GV-to-MII transition, favouring a more physiological asymmetric division (Fig. 7). Further studies will be needed to understand the mechanism of action of this catecholestrogen, although three of its described features may give us clues to explain our results. One of these refers to the fact that 2-OHE_2_ contains a phenolic functional group that has been shown, in smooth muscles, to work as a potent antioxidant eliminating free radicals [58, 59] and protecting membrane phospholipids against peroxidase [60]. Indeed, COCs cultured in the presence of antioxidants (i.e. sodium citrate, α-lipoic acid and acetyl-l-carnitine) complete the GV-to-MII transition with significant reduced number of oocytes with abnormal spindles [61]. In alternative, 2-OHE_2_ could work as an E_2_ antagonist. Earlier studies showed that culturing catfish post-vitellogenic follicles in the presence of 1–20 μM 2-OHE_2_ induced a 6–17-fold increase in the number of oocytes that resumed meiosis [22–24, 62]. This improvement correlated with increased steroid production [63, 64], inhibition of aromatase activity [65] and decreased follicular cAMP, which would result in the removal of the inhibitory effects of E_2_ on oocyte maturation and meiotic resumption. Another possible explanation on how 2-OHE_2_ improves the female gamete quality is related with its role in the production of nitric oxide, a key factor that regulates germinal vesicle breakdown and PB-I extrusion in mouse oocytes [66]. Interestingly, a recent study showed that 10 nM 2-OHE_2_ induced an increase of nitric oxide production by ovine uterine artery endothelial cells, whereas 100 nM was unable to produce this effect [67].

In conclusion, we demonstrated that, when present at 1 nM concentration during mouse COC in vitro maturation, 2-OHE_2_ has a beneficial effect on the meiotic and developmental competence of the enclosed oocytes. Our results contribute a first step to acknowledge a potential role of this estradiol metabolite during the latest stages of mammalian folliculogenesis.

## Electronic supplementary material

ESM 1 (PDF 87 kb).
