# Supplementary material for: In Vitro Maturation of Fully Grown Mouse Antral Follicles in the Presence of 1 nM 2-Hydroxyestradiol Improves Oocytes’ Developmental Competence
Source: Reprod Sci. 2020 Aug 5;28(1):121–33. doi: 10.1007/s43032-020-00276-6 (PMC7782423; doi:10.1007/s43032-020-00276-6)
Supplement: Supplementary file 1 — (PDF 87 kb). [file 43032_2020_276_MOESM1_ESM.pdf]

Table 1S. Rate of *in vitro* maturation and preimplantation embryonic development of COCs in the presence or absence of 0.002% DMSO (vehicle). The comparisons were performed within the vertical column.

Treatment	Stages of oocyte maturation and preimplantation development Number (% ± SEM)							
	**COC**	**Blocked GV or GVBD**	**Fragmented or picnotic**	**MII**	**Inseminated MII**	**2-cell**	**4-cell**	**Blastocyst***
α-MEM	134	6 (5.7 ± 2.0)	3 (1.8 ± 0.8)	126 (93.8 ± 0.5)	102	66 (64.7 ± 5.2)	35 (34.8 ± 2.1)	18 (28.3 ± 2.6)
α-MEM-DMSO	152	9 (5.9 ± 1.5)	2 (1.0 ± 0.6)	141 (93.1 ± 0.6)	121	79 (64.8 ± 3.2)	41 (34.6 ± 3.9)	22 (28.8 ± 3.9)

(*): The developmental rate was calculated based on the number of 2-cell embryos (100%).

Table 2S. Number ± SEM (*) of blastomeres forming the whole blastocyst, the inner cell mass (OCT4) or the trophectoderm (CDX2).

Medium employed (No. of Blastocysts)	DAPI	OCT4	CDX2
α-MEM (20)	34.6 ± 2.7	7.3 ± 0.6	27.3 ± 2.5
α-MEM-DMSO (19)	32.3 ± 1.6	7.4 ± 0.6	25.3 ± 1.4
